# A classroom-based test of the absolute depth theory of stereopsis

**DOI:** 10.1177/20416695251400522

**Published:** 2025-12-09

**Authors:** Robert D. McIntosh

**Affiliations:** 1 3124 University of Edinburgh, UK

**Keywords:** perception, spatial vision, stereopsis, 3D perception

## Abstract

Stereopsis, the visual experience of palpable depth and solidity, is traditionally thought to depend on the congruence or conflict amongst depth cues. But a more speculative theory is that it depends critically on being able to estimate the absolute depths of objects, and their real-world distances from us. We tested this idea in a perception class, using a picture of some plants, a cardboard box with a monocular viewing hole, and a pair of pinhole glasses. Fourteen of 16 students reported *stronger* stereopsis with pinhole viewing, contrary to the predictions of the absolute depth theory, but consistent with a traditional account. This classroom exercise offers an empirical challenge for the absolute depth theory, and a vivid teaching tool for the paradoxes of pictorial depth perception.

## How to cite this article

McIntosh, R. D. (2025). A classroom-based test of the absolute depth theory of stereopsis. *i -Perception, 16*(6), 1–4. https://doi.org/10.1177/20416695251400522

It is embarrassing to admit that I taught perception to undergraduates for nearly two decades without realising that the ‘stereo’ in stereopsis does not refer to two eyes, but comes from the Ancient Greek ‘stereós’, meaning ‘full’ or ‘solid’. Stereopsis is the qualitative visual impression of a three-dimensional world of solid objects. This linguistic root makes sense of the otherwise-counterintuitive fact that the world does not look flat when we close one eye. Even more surprising, we can sometimes see stereoscopic depth in pictures, and *this effect is stronger when viewing with one eye*.

This ‘paradoxical monocular stereopsis’, was described by Claparède, who deduced its dependence on the congruence or conflict amongst depth cues (Claparède, 1904, translated by O'Shea, 2017). When we view the world with both eyes, binocular disparities give rich depth information that is consistent with the (monocular) cues in the scene. But when we look at a *picture*, the same binocular cues specify the true *flatness* of the image, contradicting the pictorial cues. These cues to flatness do not change the magnitude of depth signalled by the pictorial cues, but they over-rule the impression that it is real, palpable depth. Closing one eye removes these conflicting binocular cues, so a stronger illusion of depth is obtained.

One hundred years ago, Ames (1925) put monocular viewing at the top of his list of methods for increasing the illusion of depth from single pictures. Schlosberg (1941) likewise enumerated ways to eliminate ‘flatness’ from pictures, including monocular viewing and looking through a tube so that the picture boundaries fall beyond the field of view. The latter effect was revisited by Vishwanath and Hibbard (2013), who confirmed that naïve observers report strong stereoscopic depth when viewing pictures monocularly through a 12–15 mm aperture. If we consider the picture boundaries as providing information that the scene is just a picture in a frame, then aperture viewing can be understood to strengthen the illusion of depth, by removing the conflicting cue to flatness.

However, Vishwanath and Hibbard (2013) interpreted this aperture effect in support of a novel and different theory of stereopsis. They proposed that stereopsis arises when we can represent the absolute depths of objects, and their real-world distances from us. The scaling of relative into absolute depths requires an estimate of the distance to a fixated object. Monocular viewing of an apertured picture not only removes cues to flatness, it also removes binocular convergence, a potential cue to the distance of the picture surface. However, it leaves accommodation unaffected, which may offer a residual distance signal in this viewing arrangement. Vishwanath and Hibbard conjectured that ‘*the brain attributes the accommodation response to the pictorial objects and assigns any associated distance information to them, thereby allowing absolute depth values to be derived and generating an impression of stereopsis*’ (p. 1683).

This speculative theory of stereopsis has been criticised on logical and theoretical grounds (Rogers, 2019), but it seems not to have been subjected to any direct test. This is surprising, because it makes an easily testable prediction: if paradoxical monocular stereopsis depends on a residual distance estimate from accommodation, then it should be reduced by viewing through a pinhole, which removes the retinal blur stimulus for accommodation. Remarking on this, Rogers stated, to the contrary, that pinhole viewing gave him a ‘*clearer and more vivid*’ impression of depth (Rogers, 2019, p. 165); and Ames (1925) similarly suggested that the impression of depth from pictures could be enhanced by viewing through a small (∼2 mm) hole. Would the same hold for less expert observers?

**Figure 1.**
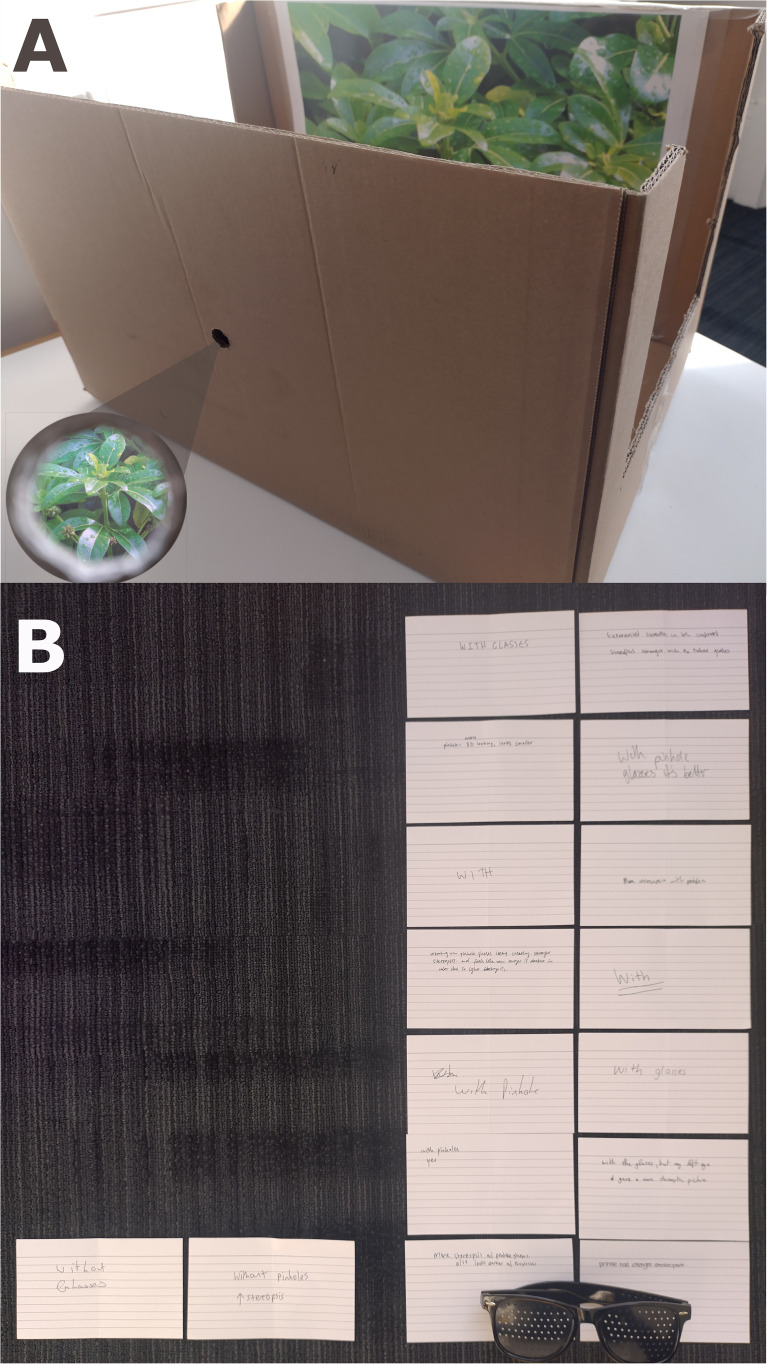
(A) Sixteen students viewed an A3 colour printout of a photo of plants through a 17 mm hole in the front of an open cardboard box. This afforded a monocular view of the picture stuck to the inside back wall, at a viewing distance of 330 mm, such that the boundaries of the picture were beyond the field of view (see inset). (B) Each student viewed the picture with and without pinhole glasses (1.3 mm holes), and wrote on a card whether they experienced stronger stereopsis with or without the glasses. Fourteen students rated stereopsis as stronger with the pinhole glasses, and only two reported the opposite impression.

The empirical test was simple enough to build into a classroom exercise. My perception students were the ideal participants, having discussed the nature of stereopsis (and the Greek root of the term), but without detailed knowledge of the absolute depth theory. On a table at the front of the room, I placed an open-top box (Figure 1A), which allowed apertured viewing of a pictorial stimulus from Vishwanath and Hibbard (2013). During a 15-minute break in a lecture, 16 students came forward to peer into the box with one eye, with and without pinhole glasses. They were encouraged to consider each view, in any order, as often as needed, to determine which gave the greater sense of stereopsis, and to write their judgement privately on a card.

This sample was more than sufficient for a clear majority (Figure 1B): 14 students rated stereopsis as stronger with the pinhole glasses (*p* = .004, two-tailed binomial test). Their perceptions, like those of the expert observers, are consistent with accommodation acting as a cue to the flatness of the picture, so that removal of this conflicting cue enhances the sense of palpable depth. But the outcome flatly (!) contradicts the absolute depth theory, according to which the removal of accommodation information should have reduced stereopsis, by removing a residual cue to distance. Whilst it would be prudent to replicate this result under formal experimental conditions, this classroom test offers an empirical challenge for the absolute depth theory, and a vivid teaching tool for the paradoxes of pictorial depth perception.
